# Brown Spider Venom Phospholipase-D Activity upon Different Lipid Substrates

**DOI:** 10.3390/toxins15020109

**Published:** 2023-01-27

**Authors:** Daniele Chaves-Moreira, Luiza Helena Gremski, Fábio Rogério de Moraes, Larissa Vuitika, Ana Carolina Martins Wille, Jorge Enrique Hernández González, Olga Meiri Chaim, Andrea Senff-Ribeiro, Raghuvir Krishnaswamy Arni, Silvio Sanches Veiga

**Affiliations:** 1 Department of Cell Biology, Federal University of Paraná (UFPR), Curitiba 81531-980, Brazil; 2 Department of Physics, Multi-User Center for Biomolecular Innovation, State University of São Paulo (UNESP), São Paulo 05315-970, Brazil; 3 Department of Structural and Molecular Biology, State University of Ponta Grossa (UEPG), Ponta Grossa 84030-900, Brazil; 4 Department of Pharmacology, University of California San Diego, La Jolla, CA 92093, USA

**Keywords:** brown spider, *Loxosceles intermedia*, venom, phospholipase-D substrate, recombinant toxin, phospholipids

## Abstract

Brown spider envenomation results in dermonecrosis, characterized by an intense inflammatory reaction. The principal toxins of brown spider venoms are phospholipase-D isoforms, which interact with different cellular membrane components, degrade phospholipids, and generate bioactive mediators leading to harmful effects. The *Loxosceles intermedia* phospholipase D, LiRecDT1, possesses a loop that modulates the accessibility to the active site and plays a crucial role in substrate. In vitro and in silico analyses were performed to determine aspects of this enzyme’s substrate preference. Sphingomyelin d18:1/6:0 was the preferred substrate of LiRecDT1 compared to other Sphingomyelins. Lysophosphatidylcholine 16:0/0:0 was preferred among other lysophosphatidylcholines, but much less than Sphingomyelin d18:1/6:0. In contrast, phosphatidylcholine d18:1/16:0 was not cleaved. Thus, the number of carbon atoms in the substrate plays a vital role in determining the optimal activity of this phospholipase-D. The presence of an amide group at C2 plays a key role in recognition and activity. In silico analyses indicated that a subsite containing the aromatic residues Y228 and W230 appears essential for choline recognition by cation-π interactions. These findings may help to explain why different cells, with different phospholipid fatty acid compositions exhibit distinct susceptibilities to brown spider venoms.

## 1. Introduction

Brown spiders are widely distributed and are represented by more than 140 species [1]. Clinical symptoms observed after a brown spider bite are referred to as Loxoscelism [2,3] and are characterized by dermonecrotic lesions with gravitational spreading and histological massive neutrophil infiltration; often referred to as necrotic or gangrenous arachnidism. In minor cases, systemic manifestations, including renal failure and hematological disturbances, such as intravascular hemolysis and hemolytic anemia, have been observed [4,5,6]. Based on proteome and transcriptome analyses, brown spider venoms mainly comprise low-molecular-mass proteins and peptides, principally in the 5–40 kDa range [7,8,9,10]. There are three classes of “highly expressed molecules” that make up approximately 95% of the toxin-encoding transcripts in the venom gland: phospholipases-D (PLD), astacin-like proteases, and low molecular mass peptides biochemically characterized as Knottins [7,8,9,10]. The study of venom compositions from brown spiders indicates that the PLD toxin family is the principal protein responsible for the clinical symptoms observed in Loxoscelism victims [11]. The characterization of *L. reclusa*, *L. arizonica*, *L. rufescens*, *L. gaucho*, *L. intermedia*, and *L. laeta* PLDs established their critical role in envenomation [12,13,14,15,16].

We have cloned and expressed seven isoforms of PLD from *Loxosceles intermedia* with different specific activities [11]. PLDs from brown spider venoms cleave sphingomyelin (SM) to form choline and ceramide 1-phosphate (C1P). Among the four major phospholipids present in cell membranes, only SM is cleaved by PLD, and although phosphatidylcholine (PC) contains the same polar head group (choline), it is not cleaved by PLD toxins [11]. This enzyme is also capable of cleaving lysophosphatidylcholine (LPC) to form choline and lysophosphatidic acid (LPA) [17]. Likewise, PLD toxins catalyze transphosphatidylation of the phosphodiester linkage between the phosphate and head groups of phospholipids, forming an alcohol and a cyclic ceramide phosphate from SM or a cyclic phosphatidic acid from LPC [18].

Regarding the catalytic mechanism, models indicate crucial interactions for molecular recognition that could stabilize the substrate within the catalytic site before eventual rearrangement due to protonation or deprotonation upon substrate binding. The enzyme could rely on a specific mechanism to promote catalysis in an adapted environment. Lajoie et al. [18] have suggested that this mechanism is a transphosphatidylation reaction.

Based on the crystal structures of PLDs from *L. laeta* and *L. intermedia* a model of the catalytic mechanism has been proposed [19,20]. The single chain PLD molecule folds to form a distorted (α/β)_8_ barrel, where the inner barrel surface is lined with eight parallel β-strands linked by short flexible loops to eight α-helices that form the outer surface of the barrel. Class I PLDs (*L. laeta*) possess a single disulfide bridge (C51 and C57) which stabilizes the catalytic loop. Class II PLDs (*L. intermedia*) contain a second disulfide bridge (C53 and C201) that links the catalytic loop to the flexible loop. This additional disulfide bridge significantly reduces the flexibility of the region around the active site and decreases the volume of the cleft, as indicated by the temperature factors [20]. The interior of the barrel is densely packed with hydrophobic amino acids. The short N-terminal section and the C-terminal extension, which contains a short α-helix, a β-strand, and a random coiled region, cap the torus of the far side of the barrel [20]. The surface loops forming the near side of the barrel are mainly hydrophobic, and a narrow cavity provides access to the catalytic site, which is characterized by a ring of negatively charged amino acids. The catalytic and Mg^2+^ co-factor binding sites are located in a shallow depression that contains H12, H47, E32, D34, and D91 residues, which are fully conserved in all PLDs from *Loxosceles* genus [19,20,21].

Based on PLDs structural analysis, H12 and H47 have been identified as the critical residues for catalysis and are assisted by a hydrogen bonds network that involves D52, N252, and D233. Moreover, the residues E32, D34, D91, and solvent molecules coordinate the stabilization of a magnesium ion that is indispensable for catalysis [19,20,21]. Recently, Vuitika et al. [22] used the recombinant PLDs from *L. intermedia*, *L. laeta*, and *L. gaucho* venoms to perform a systematic mutational and functional investigation of the residues that have been proposed to be involved in catalysis (H12A-H47A), metal-ion binding (E32A-D34A), substrate recognition (K93A, Y228A, Y229A, and W230A), and stabilization of the flexible loop (C53A-C201A). This study confirmed the roles of these residues in catalysis and biological activity. *Loxosceles* PLDs can be classified into two clades, α, and β. The α-clade members possess high catalytic activity against sphingomyelin, whereas the activity of β-clade members is more varied [14,23]. Recently, a cage consisting of three aromatic amino acids (aromatic cage) present in the catalytic loop of α-clade PLDs was suggested to be involved in the preference of such enzymes for choline head groups over ethanolamine head groups [24]. However, the specificity for substrates containing choline remains unclear. The main aim of this study was to understand how the Brown spider venom PLDs recognize substrates (sphingomyelin over other phospholipids bearing the same polar head group) and how it selects substrates with different numbers of carbon atoms in fatty acid chains of diverse substrates.

## 2. Results

### 2.1. Evaluation of the Activity of Brown Spider Recombinant PLD on Different Sphingomyelins, Lysophosphatidylcholines, and Phosphatidylcholine

**Figure 1:**
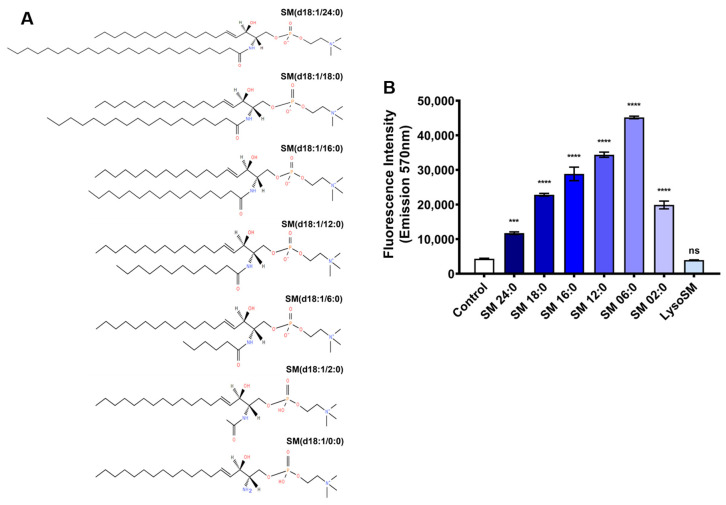
The role of the number of carbon atoms in the phospholipid chains and the amide in the Brown spider recombinant phospholipase-D (LiRecDT1) activity. (**A**) Structures of the different sphingomyelins tested. (**B**) Catalytic activity on different sphingomyelins. Control: incubation of Amplex Red reagent and substrate without the enzyme. PLD activity was measured using the Amplex Red kit (Invitrogen). LiRecDT1 toxin (1 µg) was added to different substrates (250 µM) and the Amplex Red reagent mixture. After incubation (30 min, 37 °C), fluorescence was measured by fluorimeter, excitation 540 nm, and emission 570 nm (Tecan Infinite M200). The values represent average ± SD of three independent experiments carried out in triplicate (**** *p* < 0.001 and “ns”: non-significant).

The influence of substrate size on Brown spider venom PLDs’ activity was assessed using phospholipids with acyl chains of different lengths. The brown spider recombinant PLD from *L. intermedia* venom, LiRecDT1, was cloned, expressed, and purified for all experiments [22]. Two different techniques were used to evaluate the catalytic activity of LiRecDT1 on 13 other phospholipids, where the number of carbon atoms in the fatty acid chains ranged from 0 to 24. Still, the polar head group was choline (Figure 1A). Fluorimetry and NMR techniques were used to determine the cleavage of the substrates. Figure 1B, shows that the LiRecDT1 toxin prefers sphingomyelin with six carbons in the fatty acid chain (SM 6:0) to other SMs (SM 24:0, SM 18:0, SM 16:0, SM 12:0, SM 2:0, and LSM 0:0). The enzymatic activity of LiRecDT1 increased when the number of atoms in the fatty acid chain decreased (SM 24:0 < SM 18:0 < SM16:0 < SM 12:0 < SM 6:0). However, when the fatty acid chain was very short (SM 2:0), the enzymatic activity of LiRecDT1 was reduced.

**Figure 2:**
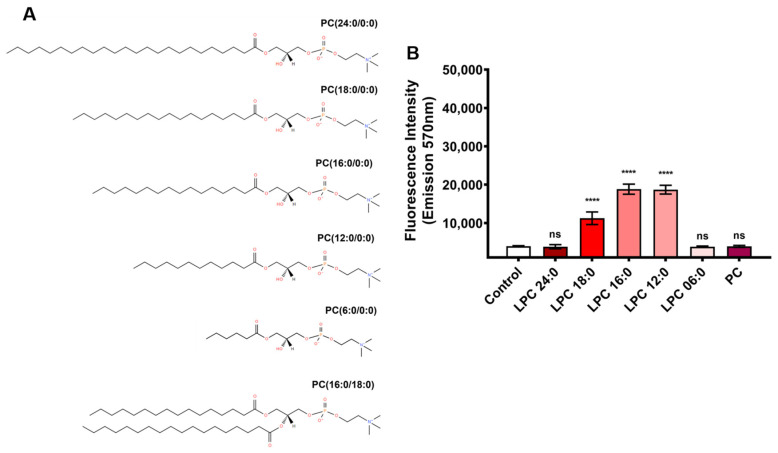
The role of the number of carbon atoms in the phospholipid chains and amide in the Brown spider recombinant phospholipase-D (LiRecDT1) activity. (**A**) Structures of the different phosphatidylcholines tested. (**B**) Catalytic activity on different phosphatidylcholines. PLD activity was measured using the Amplex Red kit (Invitrogen). LiRecDT1 toxin (1 µg) was added to different substrates (250 µM) and the Amplex Red reagent mixture. Control: incubation of Amplex Red reagent and substrate without the enzyme. After incubation (30 min, 37 °C), fluorescence was measured by fluorimeter, excitation 540 nm, and emission 570 nm (Tecan Infinite M200). The values represent average ± SD of three independent experiments carried out in triplicate (**** *p* < 0.001 and “ns”: non-significant).

Furthermore, the absence of the amide group in a sphingolipid substrate (lysosphingomyelin—LysoSM) appears to drastically affect substrate recognition by LiRecDT1 (Figure 1A,B). It is possible for the enzyme to specifically interact with the amide group, which would explain its preference for SM over PC and different LPCs (LPC 24:0, LPC 18:0, LPC 16:0, LPC 12:0, and LPC 6:0) (Figure 2A,B). PC 16:0 was not degraded, possibly because of the substitution of the amide group by an ester group in the substrate acyl chain (Figure 2B).

PC also lacks the free hydroxyl group present in the sphingolipid substrate (Figure 2A). In SM and LPC, the hydroxyl groups are at different positions, which could contribute to the observed substrate preference. This free hydroxyl group is essential for catalysis since it can function as an internal nucleophile in a transphosphatidylation reaction to form cyclic phosphate-containing products [14,18]. However, LysoSM (not tested in [18,25]), which contains this free hydroxyl group at the same position as other SMs but lacks the amide group, is not a suitable substrate. In addition, as can be observed, the 02:0 SM molecule is very similar to LysoSM (including the length of the acyl chain), but the former is a better substrate for the toxin than the latter; the main difference between both is that 02:0 SM bears an amide group and LysoSM does not. We can infer that the amino group in LysoSM (positively charged) is not a good nucleophile, and to carry out the catalytic reaction, the enzyme must deprotonate the LysoSM, which impairs the cleavage of the substrate, thus supporting the observed differences. Therefore, the amide group is crucial for substrate recognition and/or catalysis. Additionally, LPCs bearing a hydroxyl group instead of an amide group were cleaved less effectively than SM, as presented in Figure 2A,B. A sharper decrease in the number of carbon atoms in the fatty chain of LPC also caused a significant decrease in the enzymatic activity of LiRecDT1, indicating that the number of carbon atoms in the fatty acid chains of the substrate was relevant for PLD activity. It is possible to observe a preference for intermediate chain lengths in LPC and short chain lengths in SM. As SM has an acyl chain that increases the hydrophobicity, a short chain is sufficient to create the necessary hydrophobicity to favor the enzyme’s affinity. On the other hand, an intermediate chain, together with the other acyl chain of SM, may interfere with substrate accommodation inside the catalytic site. LPC does not have an additional acyl chain, so an intermediate chain length contributes to its hydrophobicity to stabilize the binding to the enzyme.

**Figure 3:**
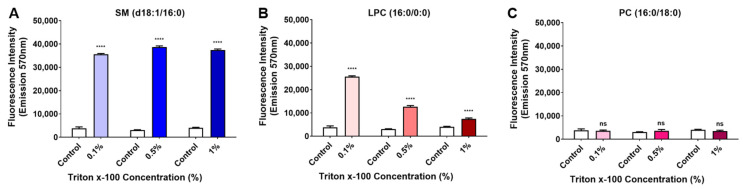
Enzymatic activity on different substrates with increasing amounts of detergent. LiRecDT1 wild type toxin (10 µg) was added to (**A**) SM, (**B**) LysoPC, and (**C**) PC substrate (250 µM) solubilized in detergent Triton-X-100 (0.1%, 0.5%, and 1%). PLD activity was measured using the Amplex Red kit (Invitrogen). Control: incubation of Am plex Red reagent mixture without the enzyme. After incubation (30 min, 37 °C), fluorescence was measured by fluorimeter, excitation 540 nm, and emission 570 nm (Tecan Infinite M200). The values represent average ± SD of three independent experiments carried out in triplicate (**** *p* < 0.001 and “ns”: non-significant).

To assess the occurrence of possible artifacts due to low lipid solubility during the assays, enzymatic activity on different substrates was performed with increasing amounts of Triton X-100. Results depicted in Figure 3 show that even with elevated concentrations of detergent (greater than the critical micellar concentration (CMC)), the profile of enzymatic activity of LiRecDT1 upon different substrates (SM, LPC, and PC) remains relatively comparable.

**Figure 4:**
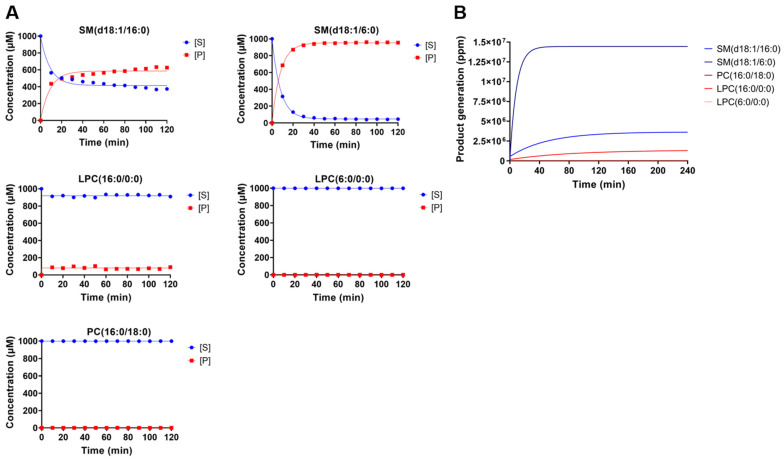
Analysis of the cleavage activity of Brown spider recombinant phospholipase-D (LiRecDT1) on different phospholipid substrates, studied by using NMR spectroscopy. (**A**) In situ NMR histograms of SM (16:0), SM (6:0), PC (16:0), LPC (16:0), and LPC (6:0) (1 mM) after recombinant phospholipase-D treatment. (**B**) Product generation plot during phospholipase-D (1 μg LiRecDT1) incubation with different phospholipids substrates in 100 mM Tris-HCl pH: 7.4, 100 mM NaCl, and 5 mM MgCl_2_ and 0.01% Triton X-100 (20 °C). Product formation was evaluated by NMR (Bruker AVANCE III HD spectrometer) every 10 min for 4 h. [S]: substrate concentration. [P]: product concentration.

In addition, to strengthen these results, product generation by LiRecDT1 on the degradation of different phospholipids was measured by NMR spectroscopy at 10 min intervals for a total of 2 h. The data presented in Figure 4 correlates with the results obtained from the fluorimetric indirect assays in Tris-Buffer (NMR spectra are available in Supplementary Figure S1). We observed that the toxin has less activity in the phosphate-buffer as the phosphate group competes with the substrate for the enzyme catalytic site. PLD toxin from *L. intermedia* shows a preference for degrading SM. Almost all SM 6:0 used in the assay (1 mM) was cleaved in 10 min, while the same amount of SM 16:0 was cleaved in 60 min. The preference of LiRecDT1 for SM 6:0 over SM 16:0 shown by fluorimetry was confirmed by NMR (see Supplementary Material Figure S2). The results also confirmed that PC 16:0 and LPC 6:0 show limited cleavage (see Supplementary Figure S2). In addition, NMR data showed that mutated isoforms of LiRecDT1 containing mutations in the histidine residues of the catalytic site (LiRecDT1-H12A and LiRecDT1-H12A-H47A) did not cleave SM 16:0, confirming the role of these amino acid residues in the catalysis (see Supplementary Figure S3). Overall, LiRecDT1 showed lower activity against LPCs and PC compared with SM.

Both techniques indicated that the amide group and the fatty acid chain length are essential for *L. intermedia* PLD recognition and activity. This is also supported by the decrease of PLD activity upon removal or substitution of the amide group by an ester group, in the case of LysoSM and PC, respectively. Additionally, when the ester group is absent (LPC), the LiRecDT1 can cleave the substrate but is less effective than SM (same size/length of the fatty acid chain). The presence of the fatty acid chain and the absence of hydrophilic groups such as hydroxyl and amine groups (LPC and LysoSM) stress the importance of a hydrophobic environment that leads to stabilization during substrate binding (reinforced by the reduction of LiRecDT1 activity when the number of carbon atoms in the fatty acid chain was significantly decreased—SM 2:0). However, since the number of carbon atoms in the fatty acid chain affects the enzyme’s substrate recognition and activity, many carbon atoms may interfere with substrate accommodation inside the catalytic site or membrane/enzyme phospholipid accessibility.

### 2.2. Kinetics Parameters of Degradation of Different Phospholipids by Brown Spider Recombinant PLD

**Figure 5:**
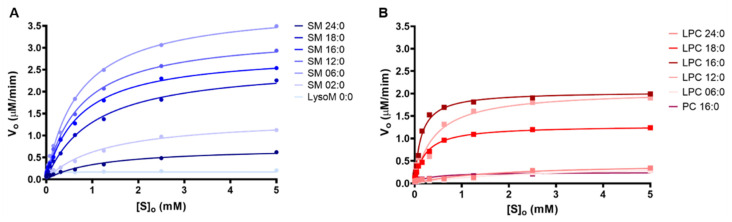
Kinetics parameters of the brown spider recombinant phospholipase-D (LiRecDT1). (**A**) Michaelis-Menten plot in the presence of different sphingomyelins (SM/LSM). (**B**) Michaelis-Menten plot in the presence of different LPCs and PCs. PLD activity was measured using the Amplex Red kit (Invitrogen). LiRecDT1 (1 µg) was added to different substrates (0.0024 to 5 mM), and the Amplex Red reagent mixture. After incubation, fluorescence was measured by fluorimeter, excitation 540 nm, and emission 570 nm (Tecan Infinite M200). The experiments were performed in 100 mM Tris-HCl pH: 7.4, 100 mM NaCl, and 5 mM MgCl_2,_ and 0.01% Triton X-100 at 37 °C. The parameters were calculated using Prism 6.0 and shown as the mean value.

All the kinetic assays were performed using the fluorimetric method using 150 nM of LiRecDT1, as described previously [26]. As shown in Figure 5 and Table 1, a substrate concentration gradient (0.0024 to 5 mM) was tested for each SM (SM 24:0, SM 18:0, SM 16:0, SM 12:0, SM 6:0, SM 2:0, and LSM 0:0), LPC (LPC 24:0, LPC 18:0, LPC 16:0, LPC 12:0, and LPC 6:0), and PC (PC 16:0) to avoid possible issues related to substrate solubility. Michaelis-Menten plots showed that among the substrates tested, the catalytic efficiency of the enzyme was highest for SM 6:0 (d18:1/6:0) and lowest for LSM (d18:1/0:0). This was probably due to the loss of the amide group in the fatty acid chain and the presence of a protonated amino group (Figure 5A,B), corroborating previous results shown in Figure 1, Figure 2 and Figure 4. The activity profiles of LiRecDT1 against other substrates missing the amide group (e.g., PC and LPC), shown as Michaelis-Menten (Figure 5), agree with the results presented in Figure 1, Figure 2 and Figure 4. Besides, they reinforce the data showing that substrates with very long (16 Carbons) or very short (2 Carbons) fatty acid chains are poorly cleaved by PLDs LiRecDT1, thus highlighting the importance of an optimal substrate accommodation inside the catalytic cleft and/or membrane accessibility.

### 2.3. Docking Analysis and MD Simulations of the Brown Spider Recombinant PLD in the Presence of Substrates

**Figure 6:**
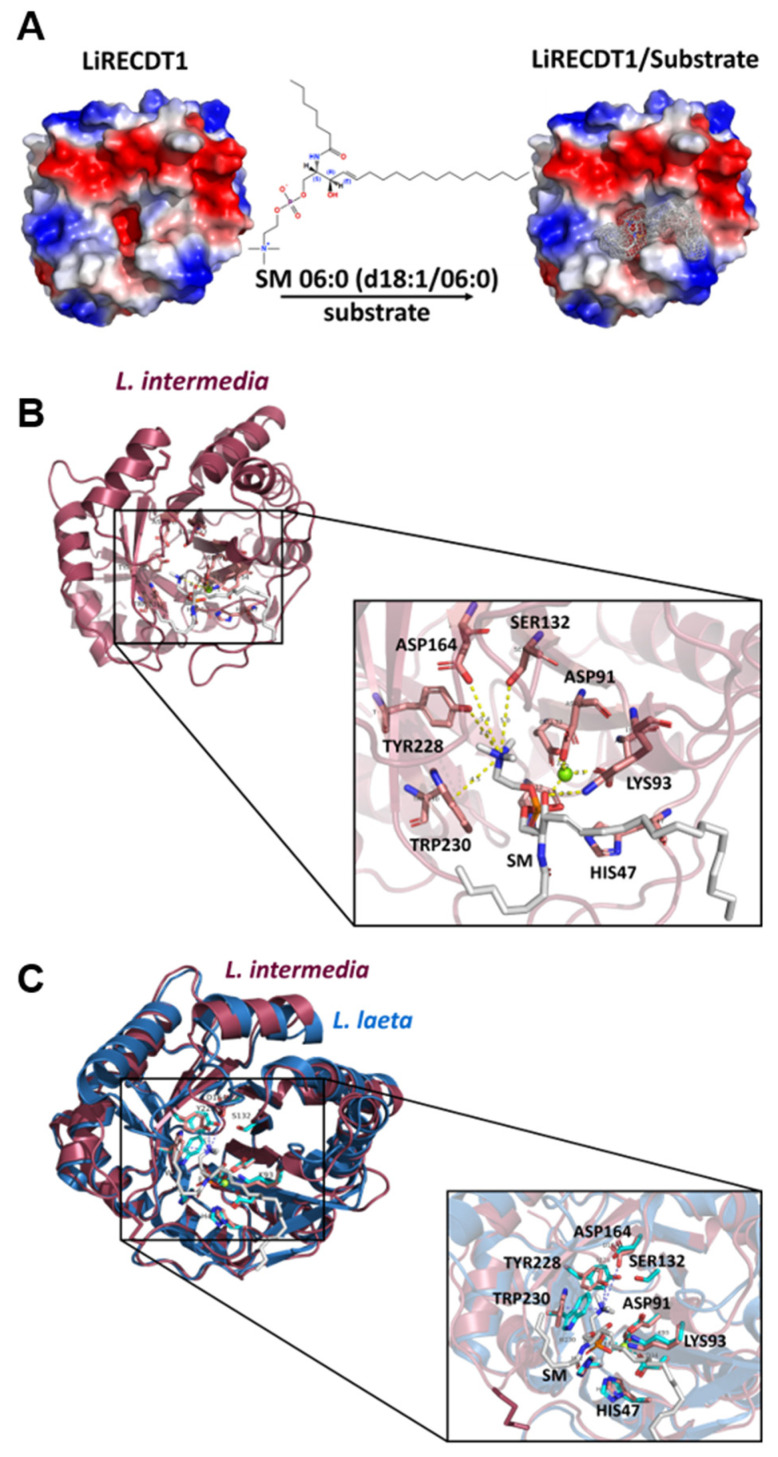
Structural models of brown spider phospholipase-D with sphingomyelin—SM 06:0 (d18:1/06:0). (**A**) Electrostatic view of LiRecDT1 indicating the position of sphingomyelin in the catalytic site of the enzyme. In blue, positive regions, red negative regions and white the hydrophobic regions. (**B**) Cartoon view of LiRecDT1 (red) bound to sphingomyelin (gray), depicting the residues important for interaction with the substrate and for catalysis. (**C**) Superposition of *L. intermedia* PLD (PDB 3RLH) with *L. laeta* Smase I (PDB 1XX1) demonstrating a high level of structural homology and sequence conservation within active site clefts. *L. intermedia* PLD has depicted as red cartoon with disulfide tethered catalytic and flexible loops highlighted in magenta. *L. laeta* SmaseI is rendered as blue cartoon with catalytic and flexible loops highlighted in cyan. Invariant active site residues of *L. intermedia* and *L. laeta* enzymes are displayed as sticks magenta and cyan with *L. intermedia* PLD numbering. The docked pose of SM 06:0 (d18:1/06:0) substrate is shown as gray sticks. Images were prepared using Pymol [29]. For interpretation of the references to color in this figure legend, the reader is referred to the web version of this article.

Based on the docking analysis and MD simulations of the crystal structure of LiRecDT1 (PDB ID: 3RLH), the substrate binding mode and the crucial residues for catalysis, were predicted. The surface electrostatic potential of LiRecDT1 reveals a deep negatively-charged pocket at the active site, responsible for coordinating the Mg^2+^ cation and accommodating the positively-charged choline moiety (Figure 6A). Docking analysis and subsequent MD simulations strongly suggest that a subsite containing aromatic amino acid residues Y228, and W230 in the LiRecDT1 appears to recognize the choline moiety via cation-π interactions (Figure 6B). This recognition mechanism of zwitterionic lipids (such as SMs), abundant in the outer leaflet of eukaryotic cells, is not well understood. However, the cation-π box can serve as the choline targeting motif [27]. It has recently been suggested that in human alkaline sphingomyelinase NPP7, the aromatic box formed by tyrosine plays a central role in choline recognition [28]. We hypothesized that the pocket containing aromatic residues is the main structural element for choline head group recognition and binding by LiRecDT1. During the MD simulations, residue W230 remains at about 4 Å from the choline group, suggesting the existence of a cation-π interaction. The residue Y228, although not so close to choline, stabilizes W230 via stacking interactions, thus indirectly participating in the accommodation of the substrate inside the active site. The phosphate group in the phospholipid is stabilized by the Mg^2+^ ion, which is coordinated by residues E32, D34, and D91 in the enzyme catalytic site (Figure 6B). The amide group of SM appeared to interact with the K93 residue, suggesting that this amino acid could participate in selecting the preferred substrate for enzyme binding (Figure 6B). This result suggests that the detrimental impact of a positively-charged amino instead of an amide moiety on LiRecDT1 activity arises from electrostatic repulsion with K93. H12 and H47 residues promote substrate hydrolysis by acid-base catalysis. The neighboring regions in contact with the aliphatic acyl chains promote the occurrence of hydrophobic interactions. When the structures of *L. intermedia* and *L. laeta* PLDs are superposed, it is possible to observe a high level of structural homology and sequence conservation within active site clefts. This similarity is maintained in the interaction profile with the substrate when the structures are docked with SM 06:0 (Figure 6C). Molecular surface representations of *L. intermedia* PLD docked with SM 06:0 confirm the results described above and reinforce the role of the aforementioned amino acid residues in the recognition and binding of LiRecDT1 with this substrate (see Supplementary Material Figure S4).

**Figure 7:**
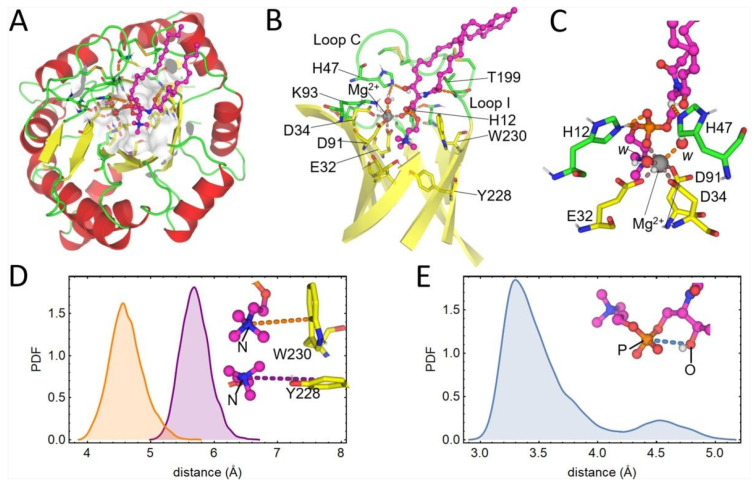
Results from 1 μs MD simulation of LiRecDT1 in complex with SM. (**A**) Upper view of the representative structure of the complex. (**B**) Side view of the complex after removing the flanking α-helices and loops outside the active site for clarity. (**C**) Zoomed-in representation of the metal-binding center of LiRecDT1, SM, and the catalytic residues H12 and H47. In all cases, SM is represented as magenta balls and sticks, while the protein is shown in the cartoon. Secondary structure elements are colored differently (α-helices in red, β-strands in yellow, and loops in green). Intermolecular hydrogen bonds prevalent during the MD simulation are depicted as orange dashed lines. The coordination bonds are represented as dark dashed lines. Active site loops I and C are labeled, and waters coordinating the metal ion are indicated with the letter w. (**D**) Distributions of distances from the choline N atom to the centers of W230 (yellow) and Y228 (purple) aromatic rings, respectively, were calculated during the MD simulation. (**E**) Same as (**D**) but for the indicated P and O atoms of SM. PDF stands for probability density function. The first 100 ns of the MD simulation were discarded before analysis.

The structure of LiRecDT1 in complex with SM generated by docking was subjected to 1 μs MD simulation in water. The central structure after clustering the trajectory with respect to the SM heavy atoms and neighboring active site residues is depicted in Figure 7. When comparing Figure 6B and Figure 7A–C, it can be noticed that the acyl chains of SM suffer significant reorganization during the MD simulation, which is likely due to the higher exposure of this substrate region to the solvent. It is worth noting that the acyl chains of PLD substrates are typically embedded inside micelles or bilayers and, therefore, the simulation of these toxins with an isolated SM molecule is expected to shed more light into the protein-lipid interactions occurring deeper into the active site. In this regard, we observed that the choline head remained tightly bound to the active site pocket of LiRecDT1 formed by the aromatic residues Y228 and W230 (Figure 7C,D). In the central structure, the Mg^2+^ ion is coordinated by six oxygen atoms, three of them are carboxylic oxygens of E32, D34 and D91, two are water oxygens and one belongs to the SM phosphate group (Figure 7C). Remarkably, we also observed that the hydroxyl group of SM remained close to the P atom along the MD simulation, thus suggesting that the cyclization of the substrate during the catalytic process is feasible [14].

## 3. Discussion

Brown spiders are dangerous arachnids that are distributed worldwide. Their propagation and adaptation, allied with their potent venom, make them a serious public health problem in several parts of the world [11]. The *Loxosceles intermedia* species is abundant and responsible for severe cases of Loxoscelism in South America [11]. These factors motivated the investigation of the main toxin responsible for the biological effects of brown spider venom.

The α-clade PLD toxins strongly prefer sphingomyelin as a substrate to LPC or PC [14,30]. This high selectivity for sphingomyelin indicates a possible role in membrane disruption [31]. The products of SM cleavage are ceramide, ceramide 1-phosphate, sphingosine, sphingosine 1-phosphate, and cyclic ceramide phosphates, depending on the phospholipase cleavage site and the presence of other enzymes such as ceramidase and sphingosine kinases. These sphingolipid metabolites are at the center of several critical biological processes, specifically in signal transduction inflammatory pathways, in which their levels change in a highly regulated temporal and spatial manner [31,32,33]. After a brown spider bite, cells are exposed to the venom toxins and susceptible to their deleterious effects. During envenomation, lipid metabolites are produced and activate several signaling cascades, such as the inflammatory pathway. The brown spider phospholipase-D studied herein was already shown to trigger dermonecrosis, neutrophil infiltration, edema, vessel permeability, hemolysis, nephrotoxicity, and lethality [11].

In this study, the fluorimetry and NMR results corroborated the generation of sphingolipid products from a solution of solubilized SM. A high preference for SM was observed, and although LPC cleavage was also observed, the efficiency was lower than 50%. The same strategies used to study phosphatidylcholine and LiRecDT1 did not show cleavage of PC, even after a longer incubation. The enzyme could distinguish between phospholipids bearing the same polar head (choline), indicating that other parts of the substrate molecule participate in the recognition and/or catalytic mechanism. Data showed that the presence of an amide group at C2 is important for *L. intermedia* PLD recognition and activity, as observed in previous studies (Lee and Lynch 2005). Notably, the carbon number in the fatty acid chains affects enzyme efficiency. The PLD toxin could cleave 80% of all SM 6:0 in 10 min, while it took 60 min to degrade the same amount of SM 16:0.

There are three main classes of lipids in cell membranes: glycolipids, phospholipids, and sterols. Moreover, there is a huge structural variety within each class, so hundreds of individual lipid species exist within each cell. Different cell types present a characteristic plasma membrane composition and a defined percentage of each phospholipid [31,34,35,36,37,38]. This could explain why the brown spider toxin can trigger different effects in different cell types.

Previous results pointed out that lipid membrane composition influences the activity of Brown spider venom PLD [39]. Furthermore, the brown spider venom phospholipase-D could select SM over other phospholipids with the same polar head. A suitable explanation for this LiRecDT1 specificity would be the amide group in the sphingolipid and its higher hydrophobicity that is absent in glycerophospholipids, which promotes the recognition and degradation of the preferred substrate.

The observed preference of LiRecDT1 for sphingolipids may have a biological significance, as sphingolipids are present at higher concentrations in the lipidome of insect prey than lysolipids, probably reflecting the brown spider evolutionary process [40]. Another interesting biological point is the significantly high percentage of phosphatidylcholine in the brown spider hemolymph and hemocytes, probably for self-protection reasons [41]. Finally, sphingomyelin is one of the most abundant phospholipid components in the outer layer of the mammalian cell plasma membrane. This explains the previously reported effects of this toxin on different cells, including endothelial, red blood cells, and fibroblasts [39].

Molecular docking of sphingomyelin showed its location inside the active site of LiRecDT1 interacting with the two catalytic H12 and H47 residues, Mg^2+^ co-factor, K93, and W230 (Figure 6 and Figure 7). The AutoDock and MD predictions agree with our experimental results and with the previously-proposed binding modes for sphingomyelin into the catalytic site of PLD St_IB1i [14]. The choline head group shows a cation-π interaction with aromatic amino acids Y228 and W230. This indicated that the cation-π interactions play an important role in the brown spider venom phospholipase-D substrate recognition. Cation-π interactions are essential for binding and catalysis by different enzymes, making tryptophan and tyrosine critical residues for binding with higher affinity and stabilizing the positive charge in transition states [42]. Also, the structural analyses that indicated the occurrence of cation-π interactions between choline and aromatic amino acid residues are corroborated by previous data that suggested the involvement of tyrosine residues through the cation-π box as the choline targeting motif [27] and by crystallographic data from sphingomyelinase NPP7, describing tyrosine residues and the cation-π box as playing a central role in choline recognition [28]. The Mg^2+^ co-factor stabilizes the phosphate moiety in the substrate while being coordinated by E32, D34, and D91 residues [19,21]. The ceramide moiety is stabilized by interactions with H12 and H47. A hydrogen bond involving the K93 amine group and the carbonyl oxygen of SM observed in the docking pose proved not to be stable during the MD simulation of the complex due to the structural flexibility of that substrate region. Nonetheless, we believe that K93 can play a crucial role in orienting the bound substrate within the active site and moderating the charge during catalysis. To evaluate the importance of the aforementioned residues in substrate binding and catalysis, Vuitika and colleagues constructed the mutants in 2016 [22]. As expected, these mutations were detrimental to sphingomyelin cleavage with significant thermodynamic changes and drastic reduction of the catalytic activity. According to Coronado and colleagues, the residue H12 is charged, and the residue H47 is neutral [21]. This protonation state stabilizes the substrate. The residues E32 and D34 coordinate the magnesium ion co-factor that stabilizes the phosphate group in the substrate.

The hydrophobic region of the SM is the long chain base (sphingosine), which attaches to a fatty acid by an amide bond to carbon 2. This region is confined within the lipid bilayer, and it is hypothesized that the LiRecDT1 interacts with the plasma membrane through three hydrophobic regions to access the phospholipid substrate. Regarding complexity, at least five different SM bases are known in mammalian cells, and more than 20 species of fatty acid (varying in chain length, degree of saturation, and degree of hydroxylation) [26]. These regions, according to their physicochemical characteristics, could lead to greater stabilization during the interaction of the toxin with the plasma membrane. In our results, the SM 6:0 perfectly fits the enzyme, probably due to the exact number of carbons in the fatty acid chain required for ideal enzyme binding and recognition.

We can conclude that sphingomyelin is the preferred lipid substrate of brown spider phospholipase-D LiRecDT1. However, lysophospholipids are also susceptible to this phospholipase-D activity, demonstrating that these enzymes cleave a broad spectrum of lipids. Point mutations in the amino acids that form the catalytic cavity indicated the importance of specific amino acid residues for substrate recognition and binding. These changes observed in the protein interactions will also be significant in drug targeting by modifying small molecules to function as PLD inhibitors, besides contributing to a better knowledge of the PLD mechanism. Until now, no specific treatment for Loxoscelism currently exists.

## 4. Materials and Methods

### 4.1. Reagents

NaCl, KCl, CaCl_2_, Na_2_HPO_4_, KH_2_PO_4_, imidazole, and agar were purchased from Merck, Germany. Tryptone and yeast extract were purchased from Himedia, India. Chloramphenicol and Ampicillin were purchased from USB, USA. Tris, IPTG, and HEPES were purchased from Sigma-Aldrich, USA. Ni^2+^-NTA agarose beads, Amplex Red Kit, and D2O were obtained from Invitrogen, USA. The synthetic sphingomyelin: SM 24:0 (d18:1/24:0), SM 18:0 (d18:1/18:0), SM 16:0 (d18:1/16:0), SM 12:0 (d18:1/12:0), SM 06:0 (d18:1/06:0), SM 02:0 (d18:1/02:0) and Egg SM 16:0 (d18:1/16:0); the synthetic lysosphingomyelin: LSM d18:1/0:0; phosphatidylcholine PC d18:1/16:0 and the synthetic lysophosphatidylcholine: LPC 24:0/0:0, LPC 18:0/0:0, LPC 16:0/0:0, LPC 12:0/0:0, and LPC 06:0/0:0 were acquired from Avanti Polar Lipids, USA.

### 4.2. Cloning, Expression, and Purification

The recombinant Phospholipase-D from *L. intermedia* venom (LiRecDT1) was cloned into a pET-14b (Novagen vector). Heterologous expression was performed in *Escherichia coli* BL21 (DE3) pLysS cells (Invitrogen, Waltham, MA, USA), as previously described [22]. Protein expression was induced by the addition of 0.05 mM IPTG (Isopropyl β-D-thiogalactoside) for 3.5 h at 30 °C (when OD550 nm > 0.5). The bacteria were chilled and disrupted by mechanical lysis. The lysed suspension was centrifuged (9000× *g*, for 30 min), and the supernatant was incubated with 1 mL Ni^2+^-NTA agarose beads for one hour at four °C. The suspensions were loaded onto a column, and the Ni^2+^-NTA agarose beads were rinsed with Wash solution pH: 8.0 (50 mM Na_2_HPO_4_, 500 mM NaCl, and 20 mM imidazole). Elution was performed using a specific buffer solution pH: 8.0 (50 mM Na_2_HPO_4_, 500 mM NaCl, and 250 mM imidazole). The collected fractions were analyzed using 12.5% SDS-PAGE, and the purest sample was dialyzed against phosphate-buffered saline. Finally, protein concentration was determined by Bradford assay [43].

### 4.3. PLD Activity

PLD activity was measured using the Amplex Red kit (Thermo Fisher Scientific, Waltham, MA, USA), a sensitive fluorogenic probe for H_2_O_2_ [22]. Briefly, LiRecDT1 cleaves SM to yield choline. Choline undergoes oxidation by choline oxidase to generate betaine and H_2_O_2_. Subsequently, H_2_O_2_ reacts with the Amplex red reagent in the presence of horseradish peroxidase and yields highly fluorescent resorufin. The assay added LiRecDT1 toxin (1 µg) to the substrate (250 µM/0.1% Triton X-100) and the Amplex red reagent mixture. After incubation (30 min at 37 °C), the resulting fluorescence was measured using a Tecan fluorimeter, Infinite M200, at an excitation wavelength of 540 nm, with emission detected at 570 nm. The same method was used to evaluate the cleavage of all other phospholipid substrates. However, the egg SM contained in the kit was replaced by other synthetic substrates: phospholipids with different numbers of carbon atoms in the fatty acid chains SM 24:0 (d18:1/24:0), SM 18:0 (d18:1/18:0), SM 16:0 (d18:1/16:0), SM 12:0 (d18:1/12:0), SM 06:0 (d18:1/06:0), SM 02:0 (d18:1/02:0), LSM (d18:1/0:0), PC 16:0 (18:0/16:0), LPC 24:0 (24:0/0:0), LPC 18:0 (18:0/0:0), LPC 16:0 (16:0/0:0), LPC 12:0 (12:0/0:0), and LPC 06:0 (06:0/0:0). All phospholipids were acquired from Avanti polar lipids, solubilized in reaction buffer (Tris-HCl 100 mM pH 7.4 containing 10 mM MgCl_2_ and 0.01% Triton X-100), at a concentration lower than their critical micellar concentration (CMC). Experiments were performed in triplicates. Alternatively, enzymatic activity determination on different substrates with increasing amounts of Triton X-100. LiRecDT1 wild-type toxin (10 µg) was added to SM, LPC, and PC substrate (250 µM) solubilized in detergent (0.1%, 0.5%, and 1%).

### 4.4. Nuclear Magnetic Resonance (NMR)

A Bruker AVANCE III HD spectrometer (Bruker, Bremen, Germany), operating at 600 MHz for ¹H and 150 MHz for ¹³C, equipped with a triple-resonance cryoprobe, pulsed-field z-gradient, was used during the analyses. Product generation by one μM of LiRecDT1 in the presence of the substrates as mentioned above, at 20 °C, in Tris-HCl 100 mM pH 7.4 containing ten mM MgCl_2_ and 0.01% Triton X-100. A series of one-dimensional ¹H were collected using excitation sculpting for water suppression. 16 scans recorded for each spectrum, and four dummy-scans were used. A sliding window of 14 ppm with an acquisition time of 1.95 s was set. A relaxation delay of 2 s was used along with a pause for home spoil recovery of 0.2 ms. Product formation was evaluated every 10 min for 2 h. The first spectrum was acquired without any enzyme. Lock, tuning, and shimming were performed. LiRecDT1 was added to the NMR tube, vortexed for 10 s, and inserted into the magnet for catalysis monitoring. Lock, tuning, and shimming were conducted again to ensure good spectrum quality.

### 4.5. Molecular Docking

Enzymes and substrates were previously submitted to AutoDock software to docking simulations [44]. Polar hydrogen molecules were incorporated into the crystal structure of LiRecDT1 (PDB code: 3RLH) during the preparation of the macromolecule, and Kollman United atom charges and atomic solvation parameters were allocated. Calculation of electron affinity and electrostatic potential was performed using Autogrid. The grid map was generated with the following features: 70 × 70 × 70 points, grid spacing of 0.625 Å, using distance-dependent dielectric constants. Molecular docking was performed using the GA-LS method. After docking, all the generated structures were assigned to clusters based on a tolerance of 1.0 Å all-atom RMSD from the lowest-energy design. Hydrogen bonding and hydrophobic interactions between docked potent agents and the macromolecule were analyzed using AutoDockTools (ADT) [44].

### 4.6. MD Simulations

The structure of LiRecDT1 in complex with SM (SM 16:0) obtained through docking was prepared for MD simulations as described below. First, the protonation state of ionizable residues at pH = 7.0 was established using the H++ web server. At the same time, SM was modeled as a zwitterion, with a positively-charged choline and a negatively-charged phosphate group. The complex was embedded in an octahedral box with edges at least 10 Å away from the solute surface. TIP3P waters and sufficient counterions (2 Cl- ions) were added to the solvation box using tleap of Amber 22 [45]. The protein and SM were parametrized with ff14SB and lipid21 force-fields, respectively [46,47]. The system was then subjected to 5000 steps of steepest descent energy minimization (EM), followed by 5000 steps of conjugate gradient EM. The resulting structure was heated in the NVT ensemble during 500 ps until reaching 298 K through a temperature linear gradient that started at 10 K. Subsequently, the NPT equilibration was conducted for 500 ps at *p* = 1 bar and T = 298 K using the Berendsen barostat and thermostat. During the two previous steps, harmonic restraints were set for the solute-heavy atoms (k = 10 kcal·mol-1·Å-2). Finally, 1 μs MD simulation was run for the equilibrated system with no restraints and in the NVT ensemble. Frames were saved every 20 ps and clustered with cpptraj of Amber 22. The cpptraj module was also employed to calculate interatomic distances along the trajectory and to perform hydrogen bond analysis. A timestep of 2 fs was established to integrate the motion equations during all MD simulations. A cutoff distance of 10 Å was set to define short-range interactions, and PME was used to handle the electrostatic interactions beyond that distance [45].

### 4.7. Statistical Analysis

The data were analyzed by analysis of variance and Tukey’s test for average comparisons in GraphPad InStat 3.0. Mean and SD values were used to plot the figures in GraphPad Prism 6.0. Results of *p* ≤ 0.001 were considered significant.

## 5. Associated Content

PDB ID: 3RLH.

PDB ID: 1XX1.

## Figures and Tables

**Table 1:** Kinetics parameters of the Brown spider recombinant phospholipase-D (LiRecDT1).

Sphingomyelins V_0_ (μM.min^−1^)		Phosphatidylcholines V_0_ (μM.min^−1^)	
SM 24:0	0.618	PC 16:0	0.212
SM 18:0	2.257	LPC 24:0	0.287
SM 16:0	2.537	LPC 18:0	1.199
SM 12:0	2.933	LPC 16:0	1.906
SM 06:0	3.493	LPC 12:0	1.805
SM 02:0	1.124	LPC 06:0	0.238
LSM 0:0	0.206		

Reaction rates (µM.min^−1^) of LiRecDT1 with various substrates: reported values are rates of reaction for the activity of LiRecDT1 over 5 mM of different substrates (30 min).

## Data Availability

Data are contained within the article and Supplementary Materials.

## Supplementary Materials

The following supporting information can be downloaded at: https://www.mdpi.com/article/10.3390/toxins15020109/s1, Figure S1: One dimensional 1H-NMR spectra of -CH2-resonance of buffers used in NMR spectroscopy experiments whose results are shown in Figures S2 and S3.; Figure S2: Analysis of the cleavage activity of Brown spider recombinant phospholipase-D (LiRecDT1) on different phospholipid substrates, studied by using NMR spectroscopy; Figure S3: Analysis of the cleavage activity of Brown spider recombinant wild-type or mutated phospholipases-D on SM 16:0, studied by using NMR spectroscopy; Figure S4: Molecular surface representation of *L. intermedia* PLD docked with sphingomyelin.
