# Supplementary material for: Brown Spider Venom Phospholipase-D Activity upon Different Lipid Substrates
Source: Toxins (Basel). 2023 Jan 27;15(2):109. doi: 10.3390/toxins15020109 (PMC9965952; doi:10.3390/toxins15020109)
Supplement: Supplementary file 1 [file toxins-15-00109-s001.zip › toxins-2111763-supplementary.pdf]

Supplementary Materials

Brown Spider Venom Phospholipase-D Activity upon Different Lipid Substrates

Daniele Chaves-Moreira [1], Luiza Helena Gremski [1], Fábio Rogério de Moraes [2], Larissa Vuitika [1],
Ana Carolina Martins Wille [3], Jorge Enrique Hernández González [2], Olga Meiri Chaim [1], Andrea Senff-Ribeiro [1], Raghuvir Krishnaswamy Arni [2] and Silvio Sanches Veiga [1,*]

[1] Department of Cell Biology, Federal University of Paraná (UFPR), Curitiba 81531-980, Brazil;

[2] Department of Physics, Multi-user Center for Biomolecular Innovation, State University of São Paulo (UNESP), São Paulo 05315-970, Brazil

[3] Department of Structural and Molecular Biology, State University of Ponta Grossa (UEPG),
Ponta Grossa *84030-900*, Brazil
* Correspondence: veigass@ufpr.br; Tel.: +55-41-3361-1776

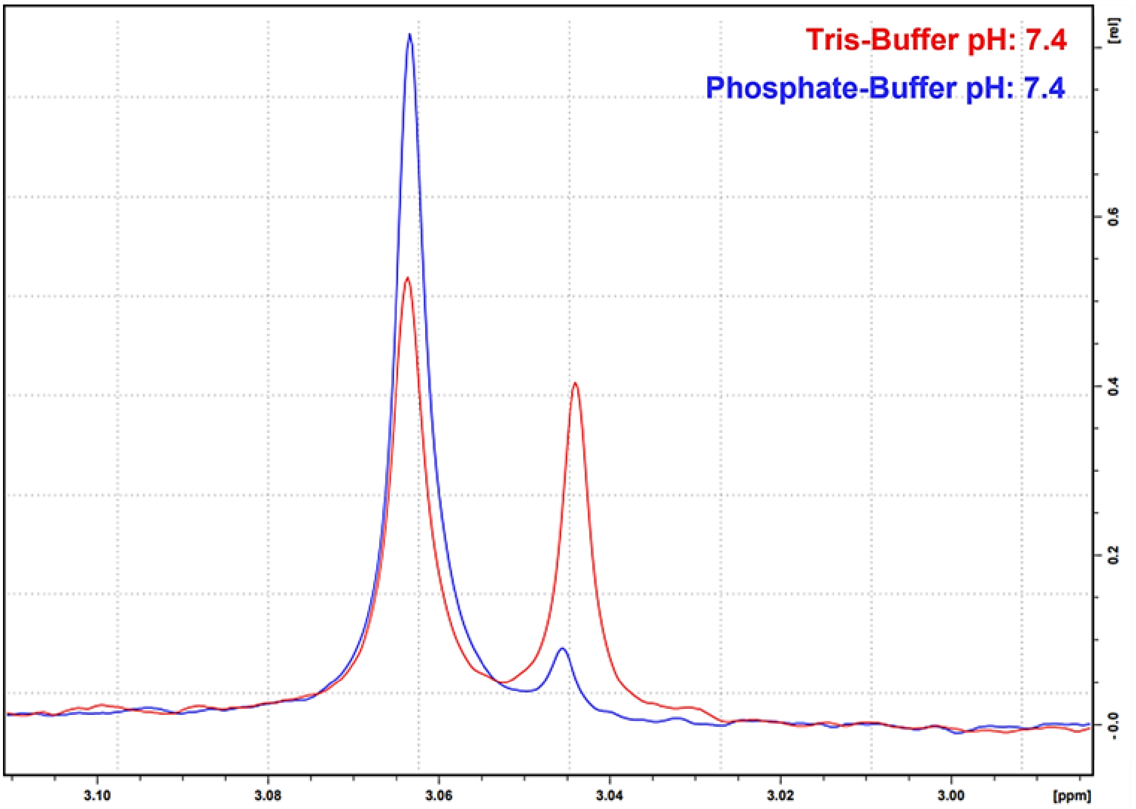

Figure S1. One dimensional 1H-NMR spectra of -CH2- resonance of buffers used in NMR spectroscopy experiments whose results are shown in Figures S2 and S3.

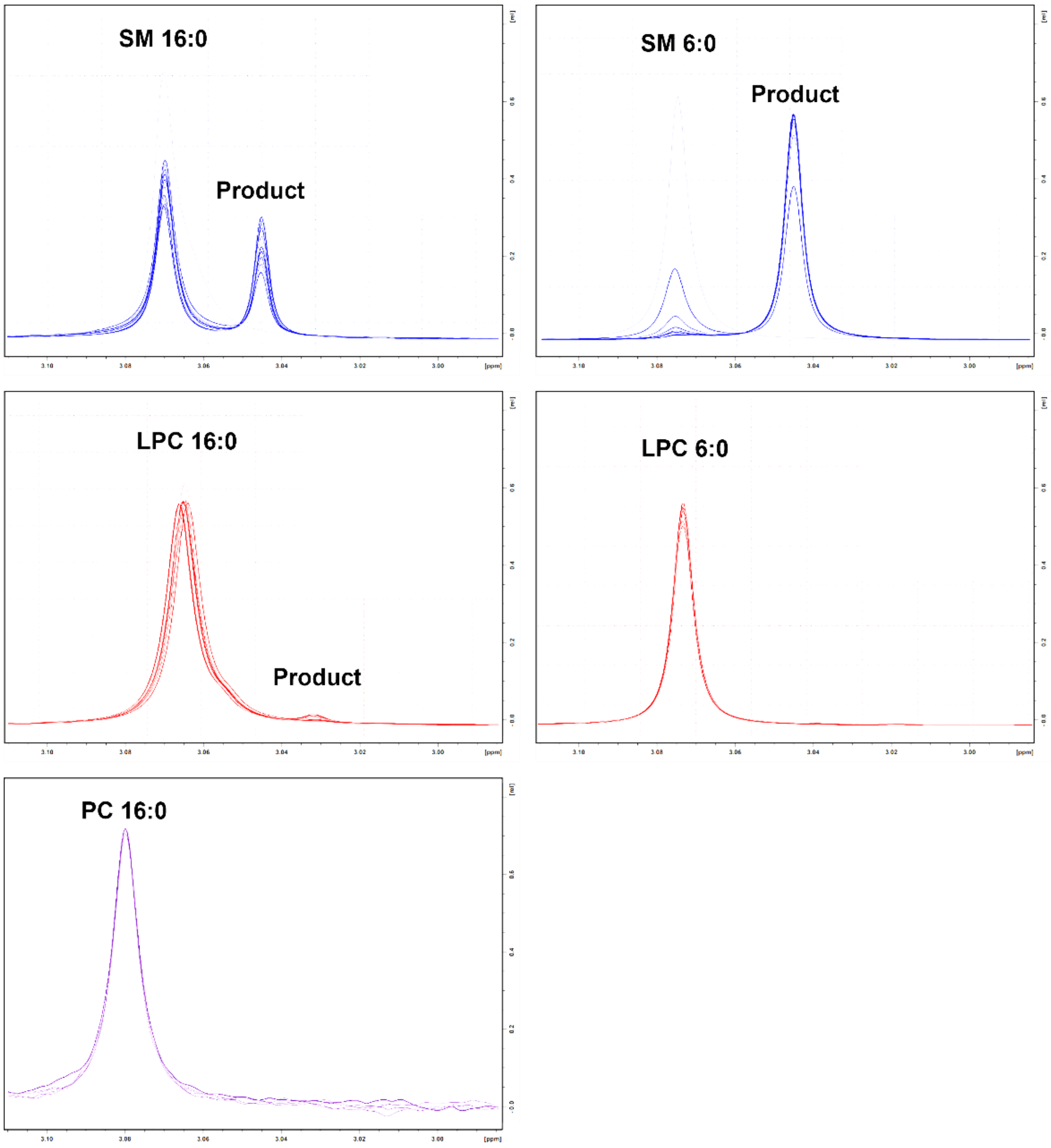

Figure S2. Analysis of the cleavage activity of Brown spider recombinant phospholipase-D (LiRecDT1) on different phospholipid substrates, studied by using NMR spectroscopy. A) One dimensional 1H-NMR spectra of -CH2- resonance of SM 16:0, SM 6:0, LPC 16:0, LPC 6:0 and PC 16:0 monitored along a 2-hour period after recombinant phospholipase-D treatment.

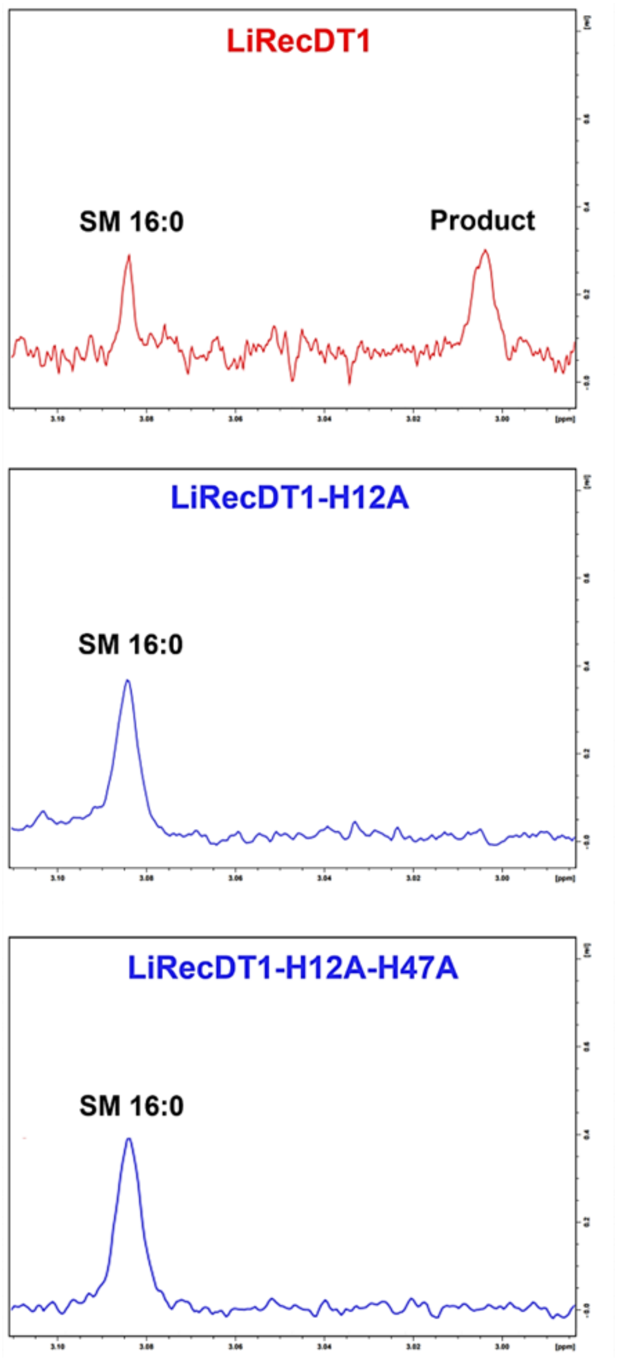

Figure S3. Analysis of the cleavage activity of Brown spider recombinant wild-type or mutated phospholipases-D on SM 16:0, studied by using NMR spectroscopy. A) One dimensional 1H-NMR spectra of -CH2- resonance of SM 16:0 incubated with LiRecDT1 (wild-type), LiRecDT1-H12A and LiRecDT1-H12A-H47A (mutations in amino acid residues of catalytic site) monitored along a 2-hour period after recombinant phospholipase-D treatment.

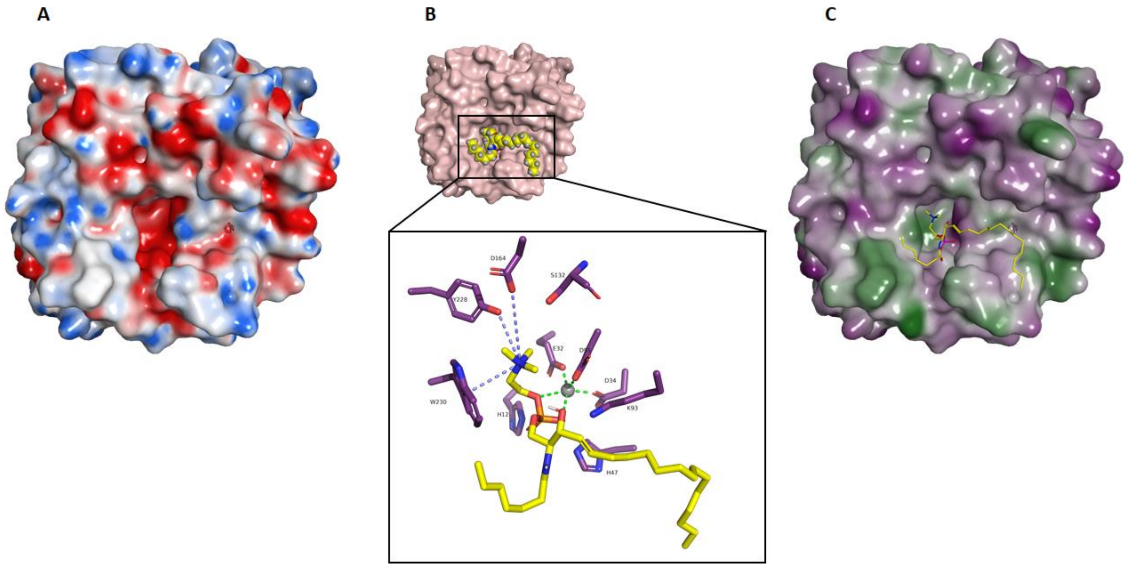

Figure S4. Structural analysis of brown spider recombinant phospholipase-D with sphingomyelin SM 06:0. (**A**) Molecular surface with electrostatics determined by Poisson-Boltzmann method showing deep electronegative substrate binding cleft of *L. intermedia* PLD. Electrostatic color map range from -40 (red) to +40 (blue). (**B**) Molecular surface representation of *L. intermedia* PLD with **SM 06:0** (d18:1/06:0) docked substrate shown as solid space-filled spheres (yellow) and close-up view of the docked pose. (**C**) Molecular surface of *L. intermedia* PLD docked with **SM 06:0** (d18:1/06:0) substrate colored by hydrophobicity (green) and hydrophilicity (purple).
